# Exploring and cataloguing the substrate space of prenyltransferases: automatic generation of SMARTS

**DOI:** 10.1186/1758-2946-6-S1-P22

**Published:** 2014-03-11

**Authors:** Jakub Gunera, Peter Kolb

**Affiliations:** 1 Department of Pharmaceutical Chemistry, Philipps-Universität Marburg, Marbacher Weg 6, 35032 Marburg, Germany

## 

Structure-activity relationship (SAR) is one of the foundational principles of biomolecular activity and states that similar molecules have similar biological activity. This work is focused on the utilization of enzymatic reactions in order to augment ligands with small apolar substituents. These modified molecules will then be employed for establishing the SAR of a ligand series.

**Figure 1:**
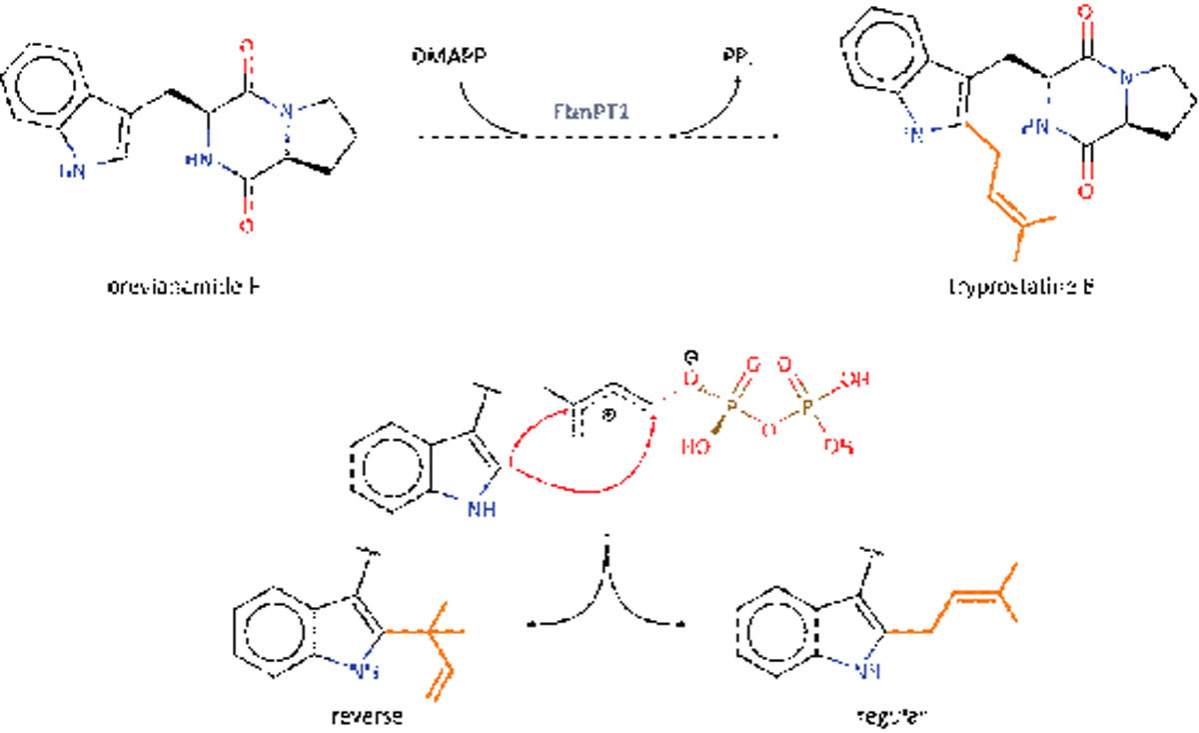
Prenyltransferase reaction exemplified by prenylation of brevianamide F by FtmPT1.

For this purpose, prenyltransferases (PT) seem like an attractive avenue. Their enzymatic attachment of the apolar prenyl moiety [1] (Figure 1) is desirable, as it does not require the precise geometric orientation of a polar interaction.

**Figure 2:**
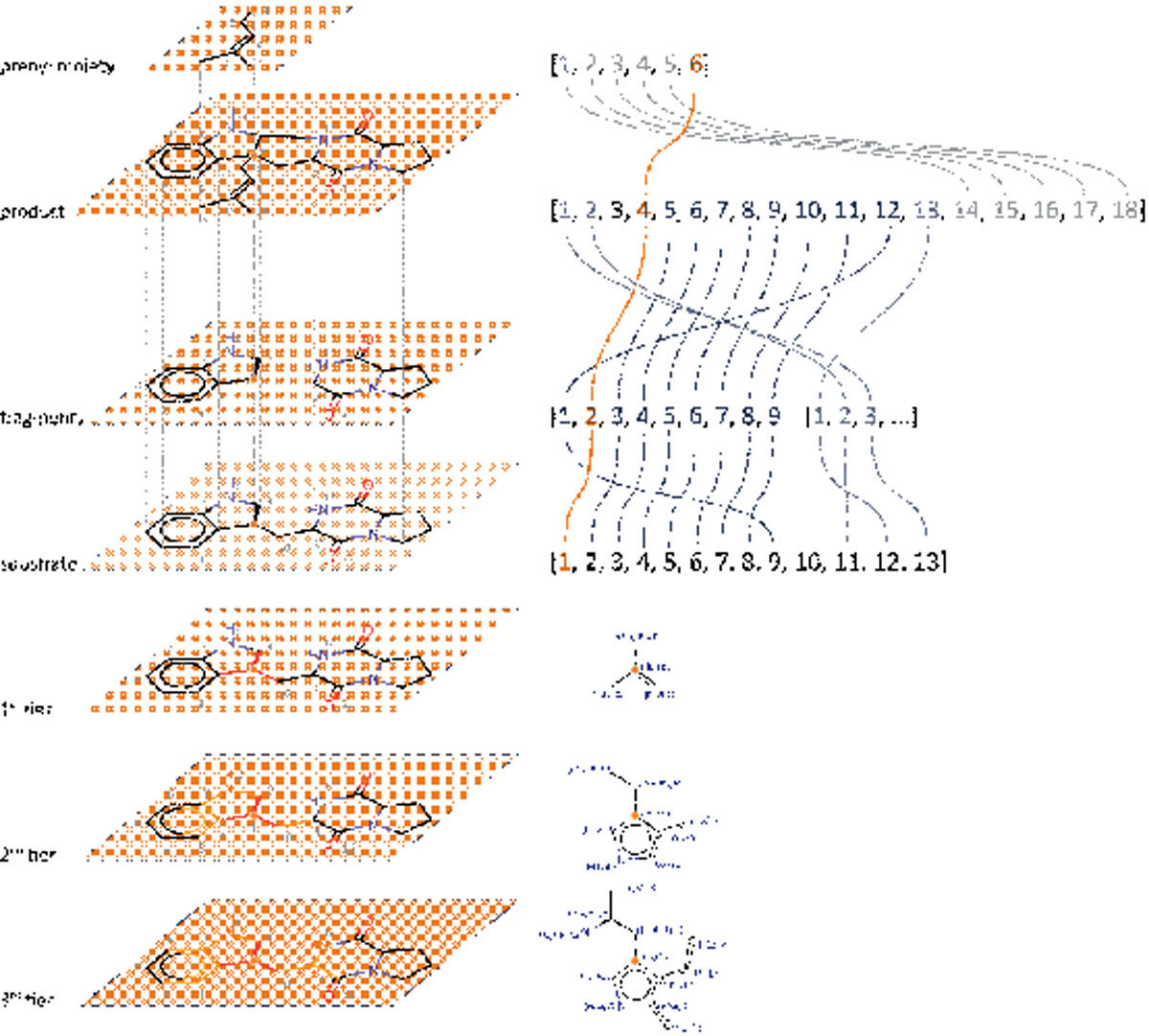
Process of generation of SMARTS substructure strings based on substrate-to-product atom mappings.

In the initial phase of this project, PTs and their reactions were catalogued and digitalized: A MySQL-database was established where reactions – extracted from primary literature – are stored as SMARTS strings. These reaction patterns will be used for generating a library of molecules accessible to prenylation. In order to automatically generate SMARTS, the atoms involved in bond cleavage and formation have to be enumerated. Fragmentation of the substrate followed by atom mapping onto the product molecule makes it possible to identify the nucleophilic atom in an automated manner (Figure 2). From the reactive atom as starting point, an atomic pattern is reconstituted within a distinct distance. This reconstitution approach ascertained the connectivity of the atoms and allowed for ring closures. The resulting SMARTS string bears atom types that can be modulated to any generality level supported by the SMARTS definitions. Studies of over 60 publications and 400 catalogued reactions revealed a huge predominance of PTs of the DMATS superfamily (**d**i**m**ethyl**a**lly**t**rypthophan **s**ynthases) and correspondingly of prenylated indole derivatives. The reactions can further be subdivided into classes of i) reverse and regular prenylation, ii) endo- and exocyclicity of the prenylation site, iii) aromaticity of the prenylation site and iv) element name of the nucleophilic atom.
